# Systematic review and meta-analysis of prolactin and iron deficiency in peripartum cardiomyopathy

**DOI:** 10.1136/openhrt-2020-001430

**Published:** 2020-10-15

**Authors:** Sinaida Cherubin, Taylar Peoples, Jessica Gillard, Samira Lakhal-Littleton, Jennifer J Kurinczuk, Manisha Nair

**Affiliations:** 1 Nuffield Department of Population Health: National Perinatal Epidemiology Unit, Oxford University, Oxford, UK; 2 Mailman School of Public Health, Columbia University, New York, New York, USA; 3 Medical Science Division, Oxford University, Oxford, Oxfordshire, UK; 4 Department of Physiology, Anatomy and Genetics, Oxford University, Oxford, Oxfordshire, UK

**Keywords:** cardiomyopathies, biomarkers, pregnancy, systematic reviews as topic, meta-analysis

## Abstract

**Objectives:**

We conducted a systematic review and meta-analysis of studies that compared levels of molecular biomarkers in women with peripartum cardiomyopathy (PPCM) to those in healthy pregnant and postpartum women to: (1) assess the evidence for prolactin (PRL) metabolism in PPCM, (2) ascertain the evidence for biomarkers of iron deficiency in PPCM, (3) identify other biomarkers associated with PPCM.

**Methods:**

We searched Medline, Embase, Cumulated Index to Nursing and Allied Health Literature (CINAHL) and the Global Health Library from inception without language restriction for studies that compared biomarkers levels in PPCM cases to healthy controls. Pooled standardised mean difference (SMD) was generated using a random effects model for the difference in levels of biomarkers.

**Results:**

Two studies assessed the association of PRL with PPCM, and reported that PPCM cases have higher levels of total PRL. No studies investigated iron metabolism in PPCM. Other biomarkers associated with PPCM included serum levels of natriuretic peptides (SMD=3.77, 95% CI 0.71 to 6.82), albumin (SMD=-0.67, 95% CI -1.01 to -0.32), C-reactive protein (SMD=1.67, 95% CI 0.22 to 3.12), selenium (SMD=-0.73, 95% CI -1.58 to 0.12), cardiac troponins (SMD=1.06, 95% CI 0.33 to 1.80), creatinine (SMD=0.51, 95% CI 0.33 to 0.69), white bloodcells (SMD=0.44, 95 % CI 0.07 to 0.82), haemoglobin (SMD=-0.45, 95% CI -0.64 to-0.26).

**Conclusions:**

More robust molecular studies are needed to explore the association between prolactin and PPCM in human subjects and to determine the extent to which iron deficiency (with or without anaemia) contributes to the risk of PPCM.

**Key questions**

**What is already known about this subject?**

- Peripartum cardiomyopathy (PPCM) is an important cardiovascular disorder of pregnancy. Biomarkers that have been associated with PPCM include increase in serum levels of prolactin and prolactin cleavage products and low levels of haemoglobin.

**What does this study add?**

- The results of this systematic review and meta-analysis of biomarkers associated with PPCM demonstrate the limited epidemiological evidence for the association with prolactin. There is some evidence for the link between anaemia and PPCM; however, it is unclear if iron deficiency contributes to this association.

**How might this impact on clinical practice?**

- Given the limited evidence of altered prolactin levels in human subjects, the current European Society of Cardiology recommendation of bromocriptine as adjunct therapy in the treatment of PPCM must be applied with caution in clinical practice.

## Introduction

Peripartum cardiomyopathy (PPCM) is defined as left ventricular heart failure (HF) presenting between the last month of pregnancy and 5 months postpartum in the absence of prior heart disease.^1^ Although PPCM is increasingly recognised as an important cause of HF in pregnant women without pre-existing cardiovascular diseases (CVD), the aetiology and pathophysiology of the disease are still being established. An investigation of molecular biomarkers associated with PPCM can provide valuable insight into the molecular profile of the disease and help improve diagnosis.

The most cited molecular hypothesis for the pathogenesis of PPCM is a prolactin (PRL)-mediated mechanism postulated by Hilfiker-Kleiner *et al*.^2 3^ Their work with rat models of PPCM showed abnormal upregulation of PRL cleaving factors (such as Cathepsin-D) compared with wild types.^2 4^ This increased expression of cleaving enzymes leads to the production of the angiostatic and proapoptotic 16 kDa isoform of PRL, which induced HF in the rats via the induction of the micro RNA-146a. Although this mechanism has been robustly shown in animal models, it is still unclear if women with PPCM also show abnormal serum levels of PRL and other markers involved in this cleavage mechanism compared with what is expected during the early postpartum period. Therefore, the epidemiological evidence for the PRL mechanism of PPCM still needs to be ascertained in humans.

On the other hand, anaemia (defined in pregnancy as haemoglobin <11 g/dL^5^) has been identified as one of the most common comorbidities of PPCM. Reported prevalence estimates of anaemia among patients with PPCM range from 16% to over 40%,^6–8^ and the associated odds of PPCM in pregnant women with anaemia are up to five folds higher than among women with normal haemoglobin concentration.^8 9^ However, the nature of this association has not been fully elucidated, so it remains unclear which of the various causes of anaemia contribute to this association. Iron deficiency anaemia is the most common nutritional deficiency observed in pregnant women, and it is also recognised as an important comorbidity observed in chronic HF. Emerging evidence suggests that iron deficiency itself may contribute to the development of cardiomyopathy even in the absence of anaemia.^10–12^ Interestingly, there is a significant drain in maternal iron that occurs during the latter part of pregnancy, which coincides with the unique onset of PPCM and hints at a possible role of maternal iron status in the pathophysiology of the disease. Therefore, it would be important to determine whether mothers who develop PPCM present with significantly different iron profiles than those who do not.

Thus, the primary objectives of this systematic review and meta-analysis were to: (1) assess the level of evidence for the PRL-cleavage mechanism in women with PPCM and (2) determine if there is any evidence of an association between biomarkers of iron deficiency and PPCM. Since this was the first comprehensive review of the literature of biomarkers in PPCM, identifying any other biomarkers associated with PPCM in pregnant or postpartum women was a secondary objective of the review.

## Methods

The protocol for this systematic review was registered in PROSPERO under the ID: CRD42019121716.

### Search strategy

The search strategy was a mix of automated and manual searches and included both published and unpublished literature. We searched Medline, Embase, CINAHL and Global Health Library for published literature. The search included a combination of broad search terms for “peripartum cardiomyopathy”, “heart failure” and “pregnancy”. We also searched the “Proquest Dissertations & Theses” database for relevant unpublished dissertations, reports and conference proceedings, as well as ongoing trials registered on www.ClinicalTrials.gov. An example of the search strategy is provided in online supplemental appendix 1.

10.1136/openhrt-2020-001430.supp1 Supplementary data

### Inclusion and exclusion criteria

All articles published since the database inception from all countries and in any languages were included. We included all observational studies (cohort, cross-sectional and case control) comparing biochemical markers in women with PPCM to women without HF, who had neither PPCM nor any other pre-existing CVD. Biomarkers were defined as cellular, biochemical or molecular factors that are measurable in biological media such as human tissue, cells or fluids. We excluded studies of non-pregnant women and women who were more than 6 months postpartum (to conform with the definition of PPCM), and studies of pregnant women with known pre-existing CVD. We reviewed and screened the reference lists of systematic reviews and meta-analyses on related subjects for relevant research articles.

### Data extraction and quality assessment

The titles and abstracts of the search results were critically screened based on the inclusion and exclusion criteria. Two reviewers (SC and TP) independently reviewed the full text of the articles generated from that screen and extracted data from the included studies on all biomarkers investigated, and the reported means and SD of the biomarker levels in all study groups (see online supplemental appendix 2 for a template of the extraction tool). Additionally, a third reviewer (JC) screened and extracted a random subset of the selected articles. We reviewed and extracted any papers that were not available in English (or any other language spoken or understood by the reviewers) with the help of native speakers. Risk of bias across studies was assessed using a modified version of the US National Heart, Lung and Blood Institute’s Quality Assessment Tool for the appropriate study design (see online supplemental appendix 3).^13^

10.1136/openhrt-2020-001430.supp2 Supplementary data

10.1136/openhrt-2020-001430.supp3 Supplementary data

### Statistical analysis

All statistical analyses in this review were performed using R V.4.0.2.

The level of inter-reviewer agreement was assessed using a Cohen’s κ statistic (a measure of chance-corrected agreement).^14^ The level of agreement between reviewers on the papers reviewed by SC and TP was ‘Moderate’ (κ=0.574; 95% CI 0.382 to 0.766). The level of agreement on the random sample of 26 papers was ‘Almost perfect’ (κ=0.845; 95% CI 0.639 to 1.051).

We conducted a meta-analysis of biomarkers with reported mean and SD values for PPCM cases and healthy controls. Given the small sample size of the included articles (median: 39 cases), we conducted the meta-analysis for biomarkers reported in at least five studies, which allowed us to have at least 150 cases per biomarker. We used Hedges’ g to compute pooled standardised mean differences (SMDs) as summary estimates for the difference in the level of these biomarkers between PPCM cases and healthy controls. Due to the statistical, clinical and methodological heterogeneity of the included studies, we fitted a random-effects model to test the differences between the groups for each biomarker identified. We used the default restricted maximum-likelihood method to estimate the level of heterogeneity.

### Patient and public involvement

Patients or the public were not involved in the design, or conduct, or reporting, or dissemination plans of our research.

## Results

### Description of the included studies

**Figure 1:**
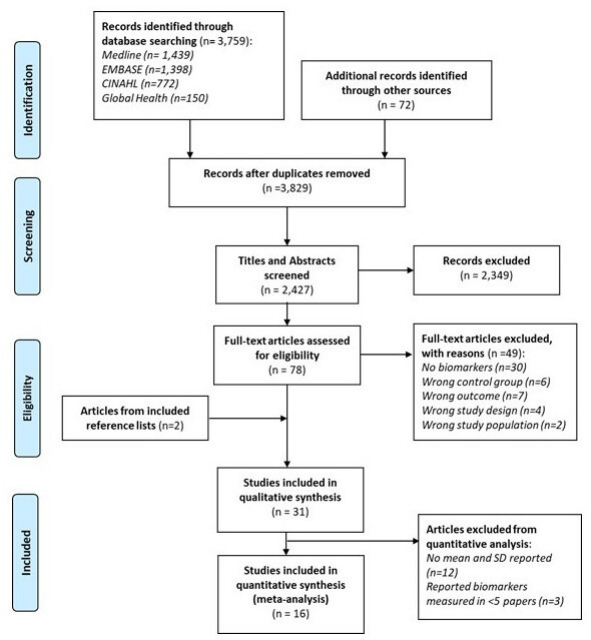
Preferred Reporting Items for Systematic Reviews and Meta-Analyses flow diagram of the study selection process.

The final search was performed on 20 February 2020. The results of the study selection process are summarised in figure 1. We screened the title and abstract of 3829 unique research articles, 80 were selected for full-text review, 31 were found eligible for inclusion and 16 of which were included in the meta-analysis. The characteristics of the included papers are listed in table 1. The number of PPCM cases studied ranged from 5 to 115 and came from 18 unique study populations. The clinical definition of PPCM used in the included articles was heterogeneous. The three main sources of heterogeneity in the diagnosis criteria for PPCM were in the window of diagnosis, the cut-off value for the left ventricular ejection fraction, and the explicit exclusion of other causes of HF.

**Table 1:** Summary of included articles

Authors (year)	Country of origin of cases	Study design	Sample size (cases) (controls)	Matching criteria	Biomarkers analysed	Gestation stage at enrolment
Adesanya *et al* (1991)^26^	Benin	Case control	14 (7) (7)	Age	Albumin; aldosterone; ANP; PRA; sodium (urine)	Postpartum cases and controls
Azibani *et al* (2020)^27^	South Africa, Germany	Case control	247 (151) (96)	Parity	Galectin-3; OPN, PINP; PIIINP; sST2	Peripartum controls
Cénac *et al* (1990)^28^	Nigeria	Case control	79 (39) (40)	None	Circulating immune complexes; heart muscle autoantibody; IgA; IgG; IgM	Postpartum controls
Cénac *et al* (1992)^29^	Niger	Case control*	71 (35) (36)	None	Selenium	Breastfeeding postpartum controls
Cénac *et al* (1996)^30^	Niger	Case control*	75 (35) (40)	Social class, age	Albumin; copper; pre-albumin; retinol binding protein; selenium; zinc	Breastfeeding postpartum controls
Cénac *et al* (2000)^31^	Niger	Case control	75 (50) (25)	None	Chlamydia (IgA; IgG; IgM)*	Not reported
Cénac *et al* (2004)^32^	Mali	Case control	56 (28) (28)	Parity	Selenium	Not reported
Cénac *et al* (2009)^33^	Niger	Case control	64 (10) (46)	None	Selenium	Postpartum controls
Ellis *et al* (2005)^34^	Haiti	Case control	24 (12) (12)	Age, parity	Anti-HSP60; Anti-HSP70; CRP; endothelin-1; GM-CSF; IFN-g; IL-10; IL-1a; IL-1b; IL-4; proBNP; sCD40L; TGF-b; TNF-a	Postpartum cases and controls
Fett *et al* (2002)^35^	Haiti	Case control	52 (18) (34)	Parity	Beta-carotene; selenium; vitamin A; vitamin B_12_; vitamin C; vitamin E	Not reported
Forster *et al* (2008)^15^	South Africa	Nested case control	63 (43) (20)	Age, pregnancy	CRP; Fas/Apo-1; IFN-g; IL-1b; IL-6; MMP-2; MMP-9; NT-proBNP; oxLDL; PRL; TGF-b1; TNF-a; VEGF	Postpartum controls
Haghikia *et al*(2013)^20^	Germany	Nested case control	134 (115) (19)	Parity	ADMA; cathepsin-D; miRNA-146a (plasma); NT-proBNP	Peripartum cases. Postpatum controls
Haghikia *et al* (2015)^36^	Germany	Case control	120 (70) (50)	Parity	AAB: MHC and TnI	Postpartum controls
Halkein *et al* (2013)^4^	Germany	Case control	56 (38) (18)	None	ERBB4 mRNA (tissue); miRNA-146a (plasma); miRNA-146a (tissue)	Postpartum controls
Hilfiker-Kleiner *et al* (2007)^2^	Germany	Case control	12 (5) (7)	Age, parity	Cathepsin-D; oxLDL; prolactin (16 kDa); prolactin (26 kDa); STAT3	Lactating postpartum cases and controls
Huang *et al* (2010)^18^	China	Case control	182 (82) (100)	Occupation, economic status, education, age difference <10 years	ADV-IgG; albumin; AMA-IgG; CBV-IgG; cTnI; hs-CRP; neutrophils; WBC	Postpartum cases and controls
Huang *et al* (2012)^37^	China	Nested case control	104 (52) (52)	Occupation, economic status, education, age difference <10 years	Albumin; cTnI; hs-CRP; NT-proBNP; WBC	Postpartum cases and controls
Karaye *et al* (2015)^38^	Nigeria	Case control	89 (39) (50)	None	Albumin; ceruloplasmin; creatinine; haemoglobin; selenium; sodium (blood)	Postpartum cases and controls
Karaye *et al* (2016)^39^	Nigeria	Case control	131 (54) (77)	None	Creatinine; potassium; sodium (blood)	Postpartum cases and controls
Liu *et al* (2014)^40^	China	Case control	73 (37) (36)	None	AAB: B1R; AAB: M2-R; NT-proBNP	Postpartum cases. Pregnant controls
McTiernan *et al* (2018)^41^	USA	Nested case control	110 (100) (10)	Postpartum days	Macrophages†; monocytes‡; NK cells§; T-cells¶	Postpartum cases and controls
Mebazaa *et al* (2017)^25^	South Africa, France	Case control	123 (83) (40)	None	Copeptin; MR-proADM; NT-proBNP; PlGF; relaxin-2; sFLT1; sST2; VEGF	Postpartum cases, pregnant controls, controls within 24 hours of delivery
Nonhoff *et al* (2017)^42^	Germany	Case control	112 (55) (57)	Gestation stage	NT-proBNP; relaxin-2	Peripartum cases. Postpartum controls. Pregnant controls.
Patten *et al* (2012)^43^	USA	Case control	50 (21) (29)	Age, parity	sFLT1	Postpartum cases. Nursing postpartum controls
Ricke-Hoch *et al* (2019)^44^	Germany	Case control	117 (64) (53)	Age	CRP; IL-1b; IL-6; NT-proBNP; PAI-1; TnT; uPA	Postpartum cases and controls
Sagy *et al* (2017)^45^	Israel	Case control	161 006(42) (160 964)	Gestational age, medical history of cardiac conditions and creatinine)	Albumin; alkaline phosphatase; ALT; ANA; AST; bilirubin; calcium; CPK; creatinine; CRP; D-dimer; GGT; glucose; haemoglobin; phosphorous; platelets; potassium; sodium (blood); TnT; TSH; urea; uric acid; WBC	Peripartum cases and controls.
Walenta *et al* (2012)^46^	South Africa, Germany	Case control	60 (24) (36)	Age, parity	EMP**; LMP; MMP; PMP††	Pregnant controls. Postpartum controls.
Wang *et al* (2018)^47^	China	Nested case control	22 (11) (11)	None	Albumin; ALT; BNP; CK-MB; creatinine; CRP; haemoglobin; TnI; WBC	Peripartum cases and controls
Xia *et al* (2016)^48^	China	Case control	76 (38) (38)	Parity	ALT; AST; B7-H1 (mRNA); C3; C4; CK-MB; creatinine; fasted blood glucose; haemoglobin; IFN-g; IgA; IgG; IgM; IL-4; PD-1 (mRNA); proBNP; T-cells; TnI	Not reported
Xia *et al* (2017)^49^	China	Case control	76 (38) (38)	Parity	Albumin; ALT; AST; creatinine; B7-H1 (mRNA); B7-H1 (protein); CK-MB; proBNP; fasted blood glucose; haemoglobinA1c; haemoglobin; hs-CRP; IFN-g; IL-4; PD-1 (mRNA); PD-1 (protein); platelets; RBC; TnI; WBC	Not reported
Yaqoob *et al* (2018)^50^	India	Case control	115 (45) (70)	None	ACE polymorphism: DD; ID; II	Peripartum cases. Postpartum controls.

*Including: *Chlamydia pneumonia*, *Chlamydia trachomatis* and *Chlamydia psittaci*.

†Macrophages included: CD14+CD16+, CD14+ CD16+ CD38+, and CD14+ CD16+ HLA-DR+.

‡Monocytes included: CD14+, CD14+ CD16−, CD14+ CD16 CD38+, and CD14+ CD16−HLA-DR+.

§NK cells included: CD3−CD56+CD16−, CD3−CD56+CD16+, CD3−CD56+CD16+CD38+, and CD3−CD56+CD16+HLA-DR+.

¶T-cells included: CD8+, CD3+, CD4+, CD3+CD4+, CD3+CD4+CD25+, CD3+CD4+CD38+, CD3 +CD4+CD8+, CD3+CD4−CD8−, CD3+CD4 CD8−CD25+, CD3+CD4 CD8−CD38+, CD3+CD4−CD8−HLA-DR+, CD3+CD56+, CD3+CD56+CD8+, CD3+CD8+, CD3+CD8+CD25+, CD3+CD8+CD38+ and CD3+CD8+HLA-DR+.

**EMPs included: CD62E+, CD144+AV+, CD31+AV+ and CD144+CD31+AV+.

††PMPs included: CD42b−AV+, CD62P+and CD62P+CD46b+AV+.

AAB:B1R, auto-antibody: β1-adrenergic receptor; AAB:MHC & TnI, auto-antibody: myosin heavy chain & troponin-1; AAB:M2R, auto-antibody: M2-muscarinic receptor; ADMA, asymmetric dimethylarginine; ADV-IgG, adenovirus immunoglobulin-G; ALT, alanine aminotransferase; AMA-IgG, anti-mitochondrial antibody immunoglobulin-G; ANA, antinuclear antibodies; ANP, Atrial natriuretic peptide; anti-HSP60, anti-Heat shock protein 60; anti-HSP70, anti-Heat shock protein 70; AST, aspartate transaminase; B7-H1, B7 homolog 1; BNP, B-type natriuretic peptide; C3, complement component 3; C4, complement component 4; CBV-IgG, coxsackie b virus immunoglobulin-G; CK-MB, creatine kinase myocardial band; CPK, creatine phosphokinase; CRP, C-reactive protein; cTnI, cardiac troponin-1; DD/ID/II, double-deletion/insertion-deletion/double-insertion; EMP, endothelial microparticles; ErbB4, erb-B2 receptor tyrosine kinase 4; Fas/Apo-1, apoptosis antigen 1; GGT, gamma-glutamyl transpeptidase; GM-CSF, granulocyte-macrophage colony-stimulating factor; hs-CRP, high-sensitivity C-reactive protein; IFN-g, interferon gamma; IgA, immunoglobulin A; IgG, immunoglobulin G; IgM, immunoglobulin M; IL-4, interleukin 4; IL-6, interleukin 6; IL-10, interleukin 10; IL-1a, Interleukin 1 alpha; IL-1b, Interleukin 1 beta; LMP, leukocyte-derived microparticles; MMP-2, matrix metalloproteinase-2; MMP-9, matrix metalloproteinase-9; MMP, monocyte-derived microparticles; MR-proADM, mid regional pro-adrenomedullin; NT-proBNP, N-terminal pro B-type natriuretic peptide; OPN, osteopontin; oxLDL, oxidised low-density lipoprotein; PAI-1, plasminogen activator inhibitor-1; PD-1, programmed cell death protein 1; PIIINP, procollagen type-3 N-terminal propeptide; PINP, procollagen type-1 N-terminal propeptide; PlGF, placental growth factor; PMP, Platelet-derived microparticles; proBNP, pro B-type natriuretic peptide; sCD40L, soluble CD40 ligand; sFTL1, soluble fms-like tyrosine kinase-1; sST2, Soluble suppression of tumorigenicity 2; STAT3, signal transducer and activator of transcription 3; TGF-b, Transforming growth factor beta; TNF-a, tumour necrosis factor alpha; TnT, troponin-T; TSH, thyroid stimulating hormone; uPA, urokinase; VEGF, vascular endothelial growth factor; WBC, white blood cells count.

We performed the quality assessment using a modified version of the National Institutes of Health Quality Assessment tool for case-control studies.^13^ The results of the quality assessment are summarised in online supplemental appendix 4. Overall, the articles included in this review had a moderate level of bias, and thus the overall quality of the sources for the review was ‘Fair’.

10.1136/openhrt-2020-001430.supp4 Supplementary data

### Summary of biomarkers associated with PPCM

**Figure 2:**
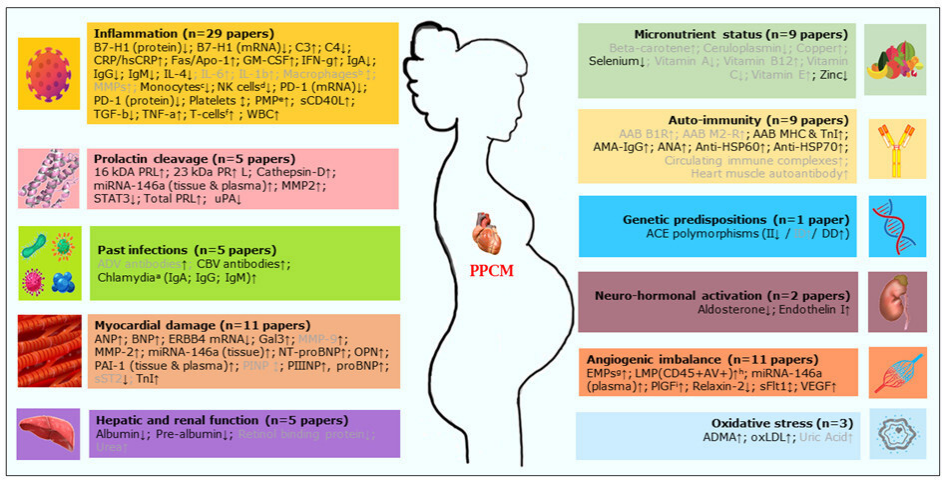
Laboratory biomarkers compared in PPCM cases and healthy controls according to their putativemechanism of effect. In grey are non-significant findings. AAB:B1R, auto-antibody: β1-adrenergic receptor; AAB:M2R, auto-antibody: M2-muscarinic receptor; AAB:MHC & TnI, auto-antibody: myosin heavy chain & troponin-1; ADMA, asymmetric dimethylarginine; ADV, adenovirus; AMA, antimitochondrial antibody; ANA, antinuclear antibodies; ANP, atrial natriuretic peptide; anti-HSP60, anti-Heat shock protein 60; anti-HSP70, anti-Heat shock protein 70; B7-H1, B7 homolog 1; C3, complement component 3; BNP, B-type natriuretic peptide; C4, complement component 4; CBV, coxsackie b virus; CRP/hs-CRP, C-reactive protein/high-sensitivity C-reactive protein; DD/ID/II, double-deletion/insertion-deletion/double-insertion; EMP, Endothelial microparticles; ErbB4, erb-B2 receptor tyrosine kinase 4; Fas/Apo-1, apoptosis antigen 1; Gal3, galectin-3; GM-CSF, granulocyte-macrophage colony-stimulating factor; IFN-g, interferon gamma; IL-4, interleukin 4; IL-6, interleukin 6; IL-10, interleukin 10; IL-1a, interleukin 1 alpha; IL-1b, interleukin 1 beta; LMP, leukocyte-derived microparticles; MMP-2, matrix metalloproteinase-2; MMP-9, matrix metalloproteinase-9; MMPs, monocyte-derived microparticles; NK cells, natural killer cells; NT-proBNP, N-terminal pro B-type natriuretic peptide; ONP, osteopontin; oxLDL, oxidised low-density lipoprotein; PAI-1, plasminogen activator inhibitor-1; PD-1, programmed cell death protein 1; PIIINP, procollagen type-3 N-terminal propeptide; PINP, procollagen type-1 N-terminal propeptide; PlGF, placental growth factor; PMP, platelet-derived microparticles; PRL, prolactin; proBNP, pro B-type natriuretic peptide; sCD40L, soluble CD40 ligand; sFTL1, soluble fms-like tyrosine kinase-1; sST2, soluble suppression of tumorigenicity 2; STAT3, signal transducer and activator of transcription 3; TGF-b, transforming growth factor beta; TNF-a, tumour necrosis factor alpha; TnI, cardiac troponin-1; uPA, urokinase; VEGF, vascular endothelial growth factor; WBC, white blood cells count. ^a^Including: *Chlamydia pneumonia*, *Chlamydia trachomatis* and *Chlamydia psittaci*. ^b^Macrophages included: CD14+ CD16+, CD14+CD16+CD38+, and CD14+CD16+HLA-DR+ ^c^Monocytes included: CD14+, CD14 +CD16−, CD14+CD16 CD38+, and CD14+CD16−HLA-DR+. ^d^PMPs included: CD42b−AV+, CD62p+and CD62p+CD46b+AV+ ^e^T-cells included: CD8+, CD3+, CD4+, CD3+CD4+, CD3+CD4+CD25+, CD3+CD4+CD38+, CD3+CD4+CD8+, CD3+CD4−CD8−, CD3+CD4 CD8−CD25+, CD3+CD4 CD8−CD38+, CD3+CD4−CD8−HLA-DR+, CD3 +CD56+, CD3 +CD56+CD8+, CD3+CD8+, CD3+CD8+CD25+, CD3+CD8+CD38+and CD3+CD8+HLA-DR+. ^f^NK cells included: CD3−CD56+CD16-, CD3−CD56+CD16+, CD3−CD56+CD16+CD38+, and CD3−CD56+CD16+HLA-DR+. ^g^EMPs included: CD62E+, CD144+AV+, CD31+AV+ and CD144+CD31+AV+ ^h^LMP: compared with postpartum controls only. ^i^PlGF: higher compared with delivery controls but lower compared with pregnant controls. ↑ Biomarkers with increased expression in the serum of peripartum cardiomyopathy (PPCM) cases compared with healthy pregnant or postpartum controls. ↓ Biomarkers with decreased expression in the serum of PPCM cases compared with healthy pregnant or postpartum controls. ↕ Biomarkers for which the direction of the difference between levels in PPCM cases and healthy pregnant or postpartum controls is conflicting.

This review included 31 case-control studies and the biomarkers were measured at a single point in time during the enrolment of participants. The studies reported the levels of 117 biomarkers in the biological media of patients with PPCM and healthy pregnant or postpartum women within the diagnostic window. Figure 2 illustrates a summary of the main biomarkers investigated according to the proposed mechanisms of effect.

#### Prolactin metabolism

Two papers measured PRL levels in PPCM cases and healthy controls. Forster *et al*^15^ reported significantly higher levels of total PRL in the serum of patients with PPCM (median=24.7 ng/mL, IQR=9.6–66.6), compared with healthy controls (median=7.4 ng/mL, IQR=2.85–18.95) (p<0.0001). Hilfiker-Kleiner *et al* detected the angiogenic 23 kDa isoform of PRL in both lactating women with PPCM and healthy lactating women.^2^ However, they found that while the angiostatic 16 kDa isoform was expressed in three of the five women with PPCM, it was barely detectable in the control group. No quantitative measurement of either isoforms was reported.

However, other biomarkers suggested to be part of the PRL-cleavage pathway to PPCM were reportedly altered in the serum of women with PPCM. Namely, levels of miRNA-146a (the main by-product of 16 kDa PRL production) were significantly increased in both the tissue and plasma of the PPCM group compared with healthy controls. Additionally, levels of both Cathepsin-D and MMP2 (factors suggested to cleave 23 kDa PRL into 16 kDa isoform) were significantly higher in cases than in controls. Finally, STAT3, which protects cardiomyocytes from an oxidative stress environment that promotes the cleavage of PRL, was expressed at lower levels in the hearts of cases compared with those of controls.

#### Iron markers

Haemoglobin was one of the most commonly reported markers in the literature. Across all the included articles, the authors found that PPCM mothers had lower levels of haemoglobin in the postpartum period compared with their healthy postpartum counterparts. However, we found no original research papers that directly assessed known markers of iron status (such as serum iron, ferritin, transferrin saturation or soluble transferrin receptors) in pregnant women with PPCM. Nevertheless, levels of other markers involved in iron homeostasis were also altered in PPCM. Levels of the cytokine IL6, which regulates the iron homeostatic hormone hepcidin, were increased in PPCM cases, although myocardial expression of STAT3 (which mediates the effect of IL6 on hepcidin) was lower than in healthy postpartum controls.^16^ Additionally, the vasoconstrictor endothelin-1 which is typically upregulated in iron deficiency^17^ was also significantly higher in PPCM cases compared with postpartum controls. Serum vitamin C, which facilitates gastrointestinal iron absorption, was lower than in healthy postpartum controls.

#### Other molecular correlates of PPCM

The most common markers studied in association with PPCM were inflammatory markers and markers of general HF, such as natriuretic peptides. N-terminal pro B-type natriuretic peptide (NT-proBNP), C-reactive protein (CRP), uric acid, antimyocardial antibodies, cardiac receptor antibodies and the D allele of the ACE gene were all identified as independent risk factors for the development of PPCM in pregnancy, although with insufficient evidence to perform a quantitative analysis (see table 2).

**Table 2:** Biomarkers identified as risk factors for peripartum cardiomyopathy

Authors (year)	Biomarker	Risk estimate (OR)	95% CI
Liu *et al* (2014)^40^	Cardiac receptor antibodies (B1R and M2R)	18.786	1.926 to 183.262
Huang *et al* (2010)^18^	Antimyocardial antibodies (AMA-IgG)	2.68	1.19 to 4.85
Huang *et al* (2012)^37^	NT-proBNP	1.92	1.12 to 4.15
Huang *et al* (2010)^18^; Huang *et al* (2012)^37^	C-reactive protein	1.86	1.08 to 4.02
Sagy *et al* (2017)^45^	Uric acid	1.3	1.049 to 1.614
Yaqoob *et al* (2018)^50^	II polymorphism of the ACE gene	0.253	0.114 to 0.558

### Quantitative analysis

We conducted a meta-analysis of papers that reported mean levels of natriuretic peptides, CRP, cardiac troponins, albumin, haemoglobin, creatinine, selenium; and white blood cells in both study groups. Generally, PPCM cases had higher serum levels of natriuretic peptides, CRP, white blood cells, cardiac troponins and creatinine, but lower levels of haemoglobin, selenium and albumin (see table 3). Forest plots for the biomarkers are provided as online supplemental materials.

10.1136/openhrt-2020-001430.supp5 Supplementary data

**Table 3:** Pooled standardised mean difference (SMD) for other biomarkers compared between peripartum cardiomyopathy cases and healthy controls

Marker	N papers	N cases	Pooled SMD	95% CI	I^2^	P value for SMD=0
Natriuretic peptides (ANP, BNP, proBNP, NT-proBNP)	10	435	3.765*	0.708 to 6.823	0.995	<0.001
Albumin	8	296	−0.665	−1.010 to −0.320	0.772	0.0012
CRP/hsCRP	7	328	1.669*	0.222 to 3.117	0.988	<0.0001
Selenium	6	165	−0.729	−1.582 to 0.124	0.931	<0.0001
Troponin	6	263	1.063	0.327 to 1.798	0.944	<0.0001
Creatinine	5	225	0.510	0.330 to 0.691	0	0.716
WBC	5	184	0.444	0.071 to 0.817	0.758	0.0098
Haemoglobin	5	168	−0.446	−0.636 to −0.256	0	0.852

*Mean values estimated from median and IQRs.

ANP, atrial natriuretic peptide; BNP, B-type natriuretic peptide; CRP/hs-CRP, C-reactive protein/high-sensitivity C-reactive protein; NT-proBNP, N-terminal pro B-type natriuretic peptide; proBNP, pro B-type natriuretic peptide; WBC, white blood cells.

## Discussion

We extracted 117 biomarkers from 31 case-control studies that compared molecular markers in PPCM to those in healthy controls. The included studied had a moderate level of bias. There is some evidence that total levels of PRL are higher in PPCM cases than in controls during the postpartum period. However, we did not find any studies that assessed iron status in PPCM. Additionally, we found that the molecular profile of PPCM is characterised by increased levels of natriuretic peptides, CRP, white blood cells, cardiac troponins and creatinine, and lower levels of haemoglobin, selenium and albumin.

### The Prolactin hypothesis for PPCM

In this review, only two studies reported levels of PRL in PPCM cases and healthy controls. One study found that women diagnosed with PPCM in the postpartum period had serum levels of PRL three times higher than the median observed in healthy controls after delivery,^18^ although no pathological cut-off has been suggested. Moreover, the authors did not report any information on the gestational age or the breastfeeding status of the study population at the time of serum analysis (both of which significantly affect PRL levels in the postpartum period). Yet, the postulated PRL cleavage mechanism is the basis for some recent clinical trials of bromocriptine for the treatment of PPCM,^19–21^ and the current ESC recommendation of bromocriptine as adjunct therapy in the treatment of PPCM.^22^ These trials are supported by evidence from animal studies, which found that the inhibition of PRL or other factors involved in the cleavage mechanism (ie, cleavage enzymes, cleavage products or downstream factors such as mi-RNA 146-a) successfully prevented the development of PPCM^4^ and associated postpartum mortality. A preliminary open-label randomised trial in ten women diagnosed with PPCM also demonstrated that reduction of PRL levels through administration of bromocriptine in addition to standard therapy was associated with greater recovery of left ventricular ejection fraction (27% to 58%; p=0.012) at 6 months compared with the control group (27% to 36%).^20^ However, the results of this review demonstrate that there are not enough studies that robustly describe the levels of biomarkers involved in this mechanism in women with PPCM. Thus, more biomolecular studies in human subjects are needed to better understand the metabolism of PRL in PPCM.

### Anaemia and iron deficiency in PPCM

Although previous studies have identified anaemia as an important comorbidity of PPCM, the current analysis of serum haemoglobin concentrations in PPCM reported in the literature does not offer strong support these findings. Indeed, the meta-analysis of mean haemoglobin concentrations in serum indicated a statistically significant but marginal pooled difference between PPCM cases compared with controls. Cases had a mean haemoglobin concentration of 10.97 g/dL, which only barely meets the WHO cut-off of 11 g/dL for the diagnosis of anaemia in pregnancy.^5^ Moreover, the search of the literature found no original papers that assessed markers of iron status in women with PPCM. Yet, past evidence has shown that even in the absence of overt anaemia, a deficiency in iron can directly undermine the myocardium’s ability to maintain contractile strength and endurance, thereby leading to left ventricular dysfunction and HF.^23 24^ This gap in the literature presents a real concern for women in low and middle-income countries, where chronic inflammation due to the high prevalence of infections, and low bioavailability of iron in the diet means that many women in these settings enter pregnancy with depleted iron stores. Thus, it is possible that a number of these women diagnosed with PPCM are developing HF secondary to iron deficiency. Therefore, future studies are needed to better understand the role of iron status in the development of PPCM, both independently and in the context of anaemia.

### Other markers of PPCM

The results of this review suggest that PPCM cases present with a molecular profile that is common to other types of cardiomyopathy. Thus, markers of general cardiac dysfunction, such as myocyte injury (cardiac troponins), myocardial stretch (natriuretic peptides), neurohormonal activation and oxidative stress, tended to be elevated in cases compared with healthy controls. Some of these markers of general HF were found to be independently associated with PPCM. However, with the exception of the genetic markers (the ACE gene), the case-control studies that measured biomarkers post event prevent us from making any inference regarding the causal effect of these biomarkers on PPCM. Moreover, because many of these biomarkers reflect general physiological events, their levels are expected to be significantly affected in a wide variety of conditions that put increased stress on the heart muscle, both in pregnant and non-pregnant populations. Thus, they offer little new or additional information on the pathophysiology specific to PPCM.

Five studies compared levels of biomarkers in PPCM to levels in other types of heart disease, and found that unlike other cardiomyopathies, PPCM exhibits increased levels of PRL, miRNA 146a and PlGF. Of note, one paper indicated that levels of PlGF and the ratio of sFlt-1 to PlGF had significant diagnostic value in distinguishing PPCM from healthy women and women with non-pregnancy-related acute HF.^25^ The specificity and sensitivity of the sFlt-1/PlGF ratio in diagnosing PPCM were 1.0 and 0.87, respectively. Comparatively, the authors found that cardiovascular markers such as NT-proBNP performed less well in the diagnosis of PPCM.^25^ Thus, these markers present interesting avenues for future research on PPCM-specific markers of disease.

### Strengths and limitations

The main limitation of this review is the strict inclusion and exclusion criteria which constrained us to only analysing papers that compared levels of biomarkers in women with PPCM to those in healthy pregnant or early postpartum women. Although there may have been other biomarkers reported in PPCM studies without a comparator group, without a comparison group we would be unable to assess, compare and pool the level of effect of the biomarkers across different studies. Additionally, a healthy control group of women without HF or CVD allows for an estimation of the baseline values of the biomarkers in a healthy pregnant population, which is important for understanding the unique biological profile of this study population. Finally, we also recognise the limitations of using an SMD as an effect estimate, as it may limit the interpretability of the results in a clinical setting.

As far as we are aware, this is the first reported comprehensive search of the literature for the molecular determinants of PPCM. By keeping the terms of the search relatively broad and manually screening for reports of biomarkers in the study of PPCM, we ensured as far as possible that all available literature on the biomolecular determinants of PPCM were captured. Moreover, the screening, data extraction and quality assessment in this review were independently performed by three reviewers, which reflects the overall robustness of the review process.

## Conclusions

The available evidence suggest that PPCM has a molecular profile that is similar to other cardiomyopathies. To date, only two studies have assessed the association of PRL with PPCM and reported that PPCM cases had higher levels of total PRL than women who did not develop PPCM. Finally, although iron metabolism is increasingly recognised as an important determinant of cardiac health, we did not find any studies that analysed iron status in PPCM. Robust population-based studies are needed to better understand the mechanisms of PRL and iron metabolism in women with PPCM.
