# Correction: *Systematic review and meta-analysis of prolactin and iron deficiency in peripartum cardiomyopathy*

**DOI:** 10.1136/openhrt-2020-001430corr1

**Published:** 2020-11-05

Cherubin S, Peoples T, Gillard J, *et al*. Systematic review and meta-analysis of prolactin and iron deficiency in peripartum cardiomyopathy. *Open Heart* 2020;7:e001430. doi:10.1136/openhrt-2020-001430

The contributors statement has been amended to remove MY, and to include MN as a contributor to the conception and design of the work.

