# Supplementary material for: Systematic review and meta-analysis of prolactin and iron deficiency in peripartum cardiomyopathy
Source: Open Heart. 2020 Oct 15;7(2):e001430. doi: 10.1136/openhrt-2020-001430 (PMC7566429; doi:10.1136/openhrt-2020-001430)
Supplement: Supplementary data [file openhrt-2020-001430supp001.pdf]

Appendix 1. Search strategy for Medline (Ovid) 1946 to present

1	*Peripartum Period/ or *Pregnant women/ or Pregnancy Complications, Cardiovascular/ or *Pregnancy Outcome/ or *pregnancy/
2	exp animals/ not humans.sh.
3	1 not 2
4	(pregnan* or labour or labor or peripartum or perinatal or prenatal or antenatal or matern* or mother* or "pregnant wom*" or "expect* mother*").ti.
5	heart failure/ or *cardiomyopathies/
6	limit 5 to pregnancy
7	6 not 2
8	*heart diseases/ or *cardiovascular diseases/
9	limit 8 to pregnancy
10	9 not 2
11	("heart failure*" or "congestive heart failure" or "chf" or "chronic heart failure" or "decompensated heart failure*" or "acute heart failure*" or "acute decompensat* heart failure*" or "peripartum cardiomyopath*" or "PPCM" or "cardiomyopath*" or "cardiac complication*" or "heart complication*" or "cardiac event" or "major cardiac event*" or "major adverse cardi* event*" or "MACE").ti,ab.
12	10 and 11
13	3 or 4
14	7 or 11 or 12
15	13 and 14
16	15 and (murine or rat or rats or mouse or mice or sheep).ti.
17	15 not 16
18	limit 17 to case reports
19	17 and ("report" or "case adj report").ti.
20	18 or 19
21	17 not 20
22	21 and (fet$ or foet* or neonat$ or newborn* or chil* or infan* or pediatri* or paediatri* or offspring*).ti.
23	21 and ((fet$ or foet* or neonat$ or newborn* or chil* or infan* or pediatri* or paediatri* or offspring*) adj1 (mother* or matern$ or wom$)).ti.
24	22 not 23
25	21 not 24
26	limit 25 to (comment or editorial or letter)
27	25 and (author* adj1 reply).ti.
28	26 or 27
29	25 not 28
