# Supplementary material for: Systematic review and meta-analysis of prolactin and iron deficiency in peripartum cardiomyopathy
Source: Open Heart. 2020 Oct 15;7(2):e001430. doi: 10.1136/openhrt-2020-001430 (PMC7566429; doi:10.1136/openhrt-2020-001430)
Supplement: Supplementary data [file openhrt-2020-001430supp002.pdf]

Appendix 2. Data extraction tool

General Information

**Date form completed** (dd/mm/yyyy)	
**Initial of reviewer extracting data**	
**Reference citation** (APA style)	
**Notes:**	

Study Eligibility

	**Eligibility criteria**	**Eligibility Criteria met?** (Y/N/Unclear)	**Location In text**
**Study Type**	Any analytical study with at least one comparator group		
**Study population**	All pregnant women or postpartum women within 5 months of delivery		
**Comparator group**	Pregnant women or postpartum women within 5 months of delivery with no pre-existing cardiovascular diseases AND without heart failure/PPCM;<br><br>OR<br><br>Pregnant women or postpartum women within 5 months of delivery with no pre-existing cardiovascular diseases AND not exposed to the Risk Factor of interest		
**Disease/Outcome of Interest**	Peripartum Cardiomyopathy		
**Outcome measures reported**	Levels and/or presence of molecular biomarkers		
**Eligibility Decision** (with reason for exclusion)			
Notes:			

DO NOT PROCEED IF STUDY IS EXCLUDED FROM REVIEW

Methods

	Description	Location in text
**Aim of the study**		
**Main outcome(s)**		
**Secondary outcome(s)**		
**Study Design**		
**Sampling Method**		
**Study period**:		
Notes:		

Population and Setting

	Description	Location in text
**Population description**		
**Cases description**		
**Comparator group description**		
**Country of origin**		
**Source/setting of the population**		
**Sample size**		
**Number of cases**		
**Definition of PPCM used:**		
**Method of PPCM Diagnosis:**		
**Exclusion criteria**		
**Gestation stage of cases at enrolment**		
**Any treatment provided?** (if yes, specify)		
**Clusters** (if applicable: # of clusters, type, # of people/cluster)		
**Withdrawals and exclusions**		
Notes:		

Variables Measured

**Demographic variable(s) measured**		
**Co-morbidities measured**		
**Risk factor(s)/Predictor(s) of HF or PCCM measured** (must have OR, RR or HR reported)		
**Laboratory biomarker(s) measured**		
Notes:		

Biomarker measurements

Biomarker 1	**Description**	**Location in text**
**Name**		
**Nature of the biomarker:** E.g. hormone, antibody, enzyme, cytokine, ionic compound etc.		
**What is the biomarker measuring?**		
**Source:** urine, blood (specify if plasma, serum, RBC etc.), saliva, etc.		
**Method of detection:** ELISA, cytometry, western blot etc.		
**Unit of measurement**		
**Time point measured**		
Notes:		

Results and findings

Biomarker 1	**Description**	**Location in text**
**Name**		
**Measurement reported:** mean, median, change in mean/median		
**Value of the measurement reported (Cases)**		
**Value of the measurement reported (Controls)**		
**Overall Significance** (95% CI and/or p-values)		
**Value of the measurement reported (clusters)** if applicable		
**Clusters Significance** (95% CI and/or p-values)		
**Key conclusions of study authors**		
Notes:		
