# Supplementary material for: Systematic review and meta-analysis of prolactin and iron deficiency in peripartum cardiomyopathy
Source: Open Heart. 2020 Oct 15;7(2):e001430. doi: 10.1136/openhrt-2020-001430 (PMC7566429; doi:10.1136/openhrt-2020-001430)
Supplement: Supplementary data [file openhrt-2020-001430supp003.pdf]

Appendix 3. Quality assessment tool for case-control studies*

*Modified from the Study Assessment Tool of the National Heart, Lung and Blood Institute[13]

General Information

**Date form completed** (dd/mm/yyyy)	
**Initial of reviewer completing the Q&A**	
**Reference citation**	

Summary of risk of bias

Risk of bias Domain	Definition	Yes/No
Research question & study population	Can we be confident that the researchers had a well-defined study goal and population?	
Control group selection	Can we be confident that the control group is a representative sample of the general population?	
Case definition	Can we be confident that the case definition did not lead to misclassification on the target condition?	
Assessment of exposure	Can we be confident in the conduct and/or interpretation of the biomarker results?	
Confounding	Can we be confident that the authors accounted for other relevant factors that could potentially explain the association between exposure and outcome.	

Overall quality Rating (Poor/Fair/Good):________________________

**Criteria**	**Yes/No/NA/Unclear (cd, nr)**	**Text location**
Domain 1: Research question & study population		
1. Was the research question or objective in this paper clearly stated and appropriate?		
2. Was the study population clearly specified and defined?		
3. Did the authors include a sample size justification?		
Domain 1 Notes:		
Domain 2: Control group selection		
4. Were controls selected or recruited from the same or similar population that gave rise to the cases (including the same timeframe)?		
5. If less than 100 percent of eligible cases and/or controls were selected for the study, were the cases and/or controls randomly selected from those eligible?		
6. Was there use of concurrent controls?		
Domain 2 Notes:		
Domain 3: Case definition		
7. Were the definitions, inclusion and exclusion criteria, algorithms or processes used to identify or select cases and controls valid, reliable, and implemented consistently across all study participants?		
8. Were the cases clearly defined and differentiated from controls?		
Domain 3 Notes:		
Domain 4: Assessment of exposure/biomarker(s)		

9. Where possible, were the investigators able to confirm that the biomarker exposure occurred prior to the development of the condition or event that defined a participant as a case?		
10a. Were the biomarker measurements clearly defined?		
10b. Were biomarker measurements valid?		
10c. Were biomarker measurements reliable?		
10d. Were the biomarker measurements implemented consistently across all study participants? (including the same time period)		
11. Were the assessors of exposure blinded to the case or control status of participants?		
Domain 4 Notes:		
Domain 5: Confounding		
12. Were key potential confounding variables measured and adjusted statistically in the analyses? If matching was used, did the investigators account for matching during study analysis?		
Domain 5 Notes:		
