# Supplementary material for: Systematic review and meta-analysis of prolactin and iron deficiency in peripartum cardiomyopathy
Source: Open Heart. 2020 Oct 15;7(2):e001430. doi: 10.1136/openhrt-2020-001430 (PMC7566429; doi:10.1136/openhrt-2020-001430)
Supplement: Supplementary data [file openhrt-2020-001430supp004.pdf]

Appendix 4. Result of the quality assessment for the included studies by assessment tool criteria

	Bias criteria*															
**First author (Year)**	1	2	3	4	5	6	7	8	9	10.a	10.b	10.c	10.d	11	12	**Overall quality**
Adesanya et al. (1991)	Y	N	N	U	U	U	N	Y	N	Y	Y	Y	Y	U	N	Poor
Azibani et al. (2020)	Y	Y	N	Y	U	Y	Y	Y	NA	Y	Y	U	U	U	N	Fair
Cenac et al. (1990)	Y	N	N	Y	U	Y	N	Y	NA	Y	N	N	Y	U	N	Poor
Cenac et al. (1992)	Y	Y	N	Y	U	Y	U	Y	NA	Y	Y	Y	Y	U	N	Good
Cenac et al. (1996)	Y	Y	N	Y	U	Y	Y	Y	N	Y	Y	Y	Y	N	N	Fair
Cenac et al. (2000)	Y	N	N	Y	U	Y	N	Y	Y	Y	Y	Y	Y	U	N	Poor
Cenac et al. (2004)	Y	Y	N	N	U	N	U	Y	NA	Y	Y	Y	N	N	Y	Fair
Cenac et al. (2009)	Y	Y	N	Y	U	Y	Y	Y	NA	Y	U	U	Y	U	N	Fair
Ellis et al. (2005)	Y	Y	N	Y	U	U	U	Y	NA	Y	Y	Y	Y	U	NA	Good
Fett et al. (2002)	Y	Y	N	Y	Y	U	U	Y	NA	Y	Y	Y	Y	U	N	Good
Forster et al. (2008)	Y	Y	N	Y	U	Y	U	Y	NA	Y	Y	Y	Y	U	N	Good
Haghakia et al. (2013)	N	Y	N	U	U	U	Y	Y	NA	Y	Y	Y	Y	U	NA	Fair
Haghakia et al. (2015)	Y	Y	N	U	U	U	Y	Y	NA	Y	Y	Y	Y	U	N	Fair
Halkein et al. (2013)	N	N	N	U	U	U	U	Y	NA	Y	Y	U	Y	U	N	Poor
Hilfier-Kleiner et al. (2007)	N	N	N	U	U	U	U	Y	Y	Y	Y	U	Y	U	N	Poor

Huang et al. (2010)	N	Y	N	Y	Y	Y	U	Y	N	Y	Y	U	Y	U	Y	Fair
Huang et al. (2012)	Y	Y	N	U	U	Y	U	Y	N	Y	Y	Y	Y	U	Y	Fair
Karaye et al. (2015)	Y	Y	N	Y	N	Y	Y	Y	U	Y	N	U	Y	U	Y	Fair
Karaye et al. (2016)	Y	Y	N	Y	U	Y	Y	Y	N	Y	Y	Y	Y	U	NA	Good
Liu et al. (2014)	Y	Y	N	U	U	U	U	Y	NA	Y	U	U	Y	U	Y	Fair
McTiernan et al. (2018)	Y	Y	N	U	U	U	U	Y	NA	Y	U	U	Y	U	N	Poor
Mebazaa et al. (2017)	Y	N	N	U	U	Y	U	Y	NA	Y	Y	Y	Y	Y	NA	Good
Nonhoff et al. (2017)	Y	Y	N	Y	U	Y	Y	Y	NA	Y	Y	U	Y	U	N	Good
Patten et al. (2012)	Y	N	N	U	U	Y	U	Y	NA	Y	Y	Y	Y	U	N	Poor
Ricke-Hoch et al. (2019)	Y	Y	N	U	U	Y	Y	Y	Y	Y	Y	U	Y	U	N	Fair
Sagy et al. (2017)	Y	Y	N	Y	NA	Y	Y	Y	U	N	U	U	Y	U	Y	Fair
Walenta et al. (2012)	Y	Y	N	U	U	U	U	Y	NA	Y	U	Y	Y	Y	N	Fair
Wang et al. (2018)	Y	Y	N	Y	Y	Y	Y	Y	Y	Y	Y	U	Y	U	N	Good
Xia et al. (2016)	Y	Y	N	U	U	Y	Y	Y	NA	Y	Y	Y	Y	U	N	Good
Xia et al. (2017)	Y	Y	N	U	U	Y	Y	Y	NA	Y	Y	Y	Y	U	N	Good
Yaqoob et al. (2018)	Y	Y	N	Y	U	Y	Y	Y	Y	Y	Y	U	Y	U	NA	Good

Legend:

Y: Yes (meets criteria), N: No (does not meet criteria), U: Unclear, NA: Not Applicable

*Bias criteria
1: Research question clearly stated; **2:** Study population specified and defined; **3:** Sample size justified; **4:** Case and control groups recruited from the same population; **5:** Random selection of study participants; **6:** Enrolment of concurrent controls; **7:** Inclusion and exclusion criteria prespecified and applied uniformly; **8:** Clear definition of cases and controls; **9:** Exposure assessed prior to outcome measurement; **10.a:** Clear definition of biomarker assessment methods; **10.b:** Valid biomarker assessment; **10.c:** Reliable biomarker assessment; **10.d.:** Biomarker assessment performed uniformly across study groups; **11:** Blinding of exposure assessors; **12:** Adjustment for confounding.
