# Supplementary material for: Systematic review and meta-analysis of prolactin and iron deficiency in peripartum cardiomyopathy
Source: Open Heart. 2020 Oct 15;7(2):e001430. doi: 10.1136/openhrt-2020-001430 (PMC7566429; doi:10.1136/openhrt-2020-001430)

S.1 Forest plot of the SMD in Natriuretic Peptides between PPCM cases and healthy controls

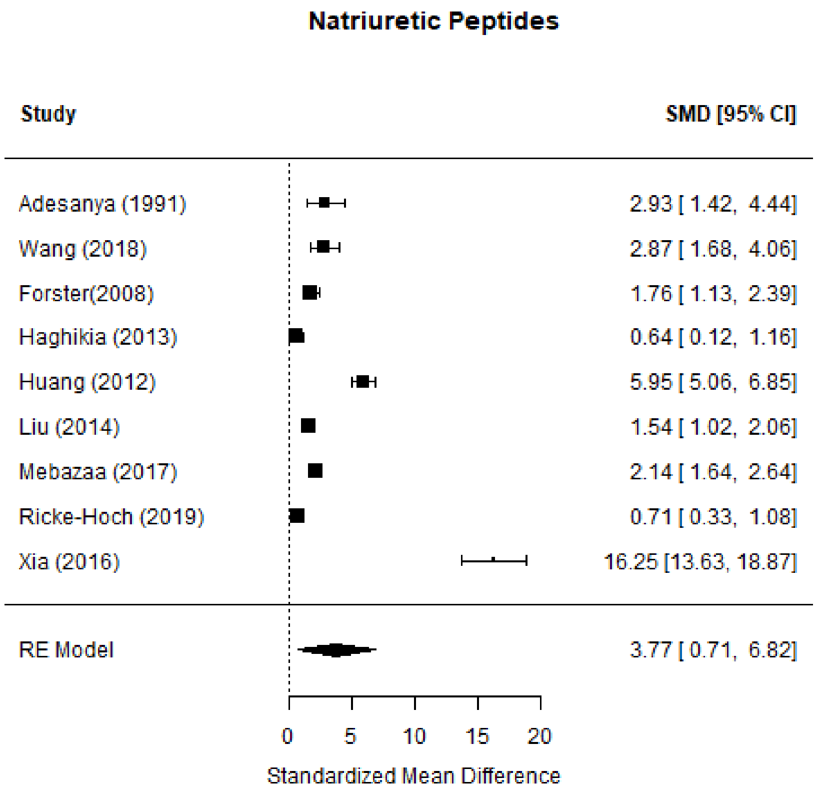

S.2 Forest plot of the SMD in Albumin between PPCM cases and healthy controls

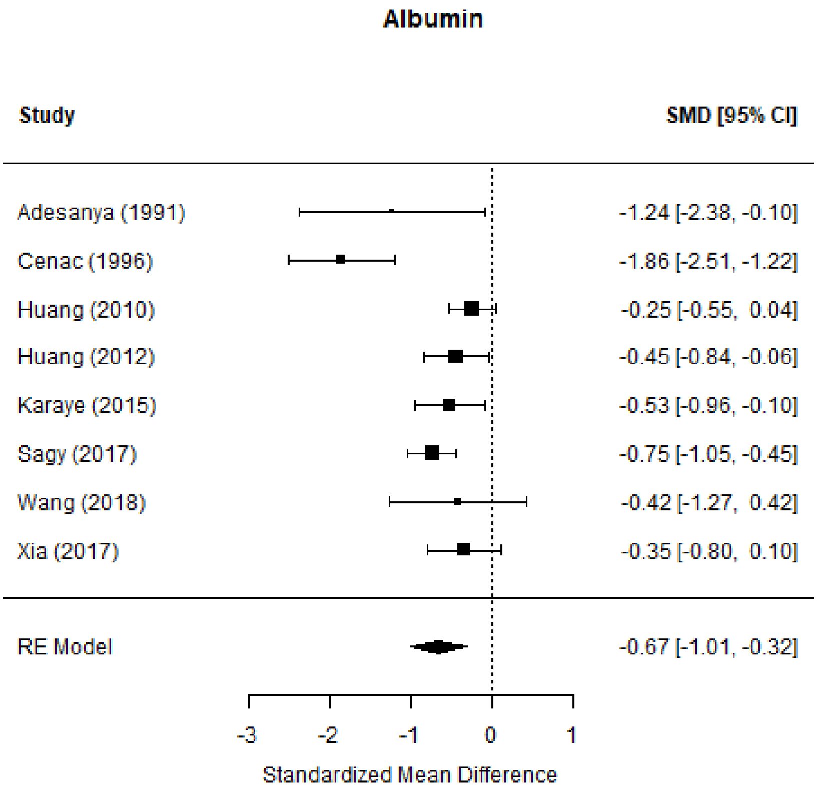

S.3 Forest plot of the SMD in CRP/hs-CRP between PPCM cases and healthy controls

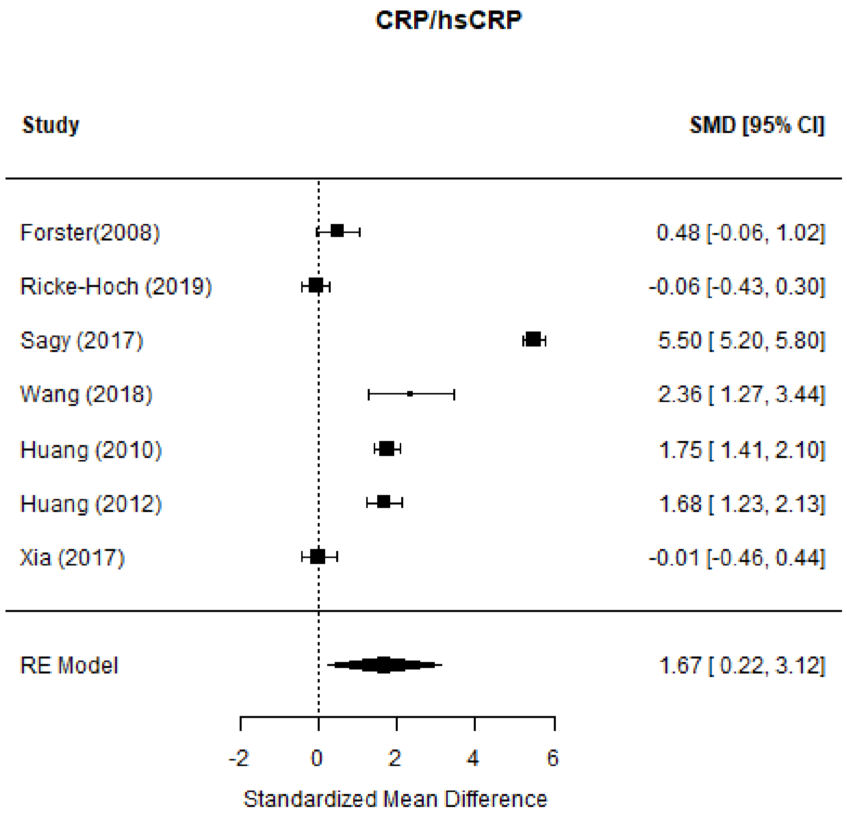

S.4 Forest plot of the SMD in Selenium between PPCM cases and healthy controls

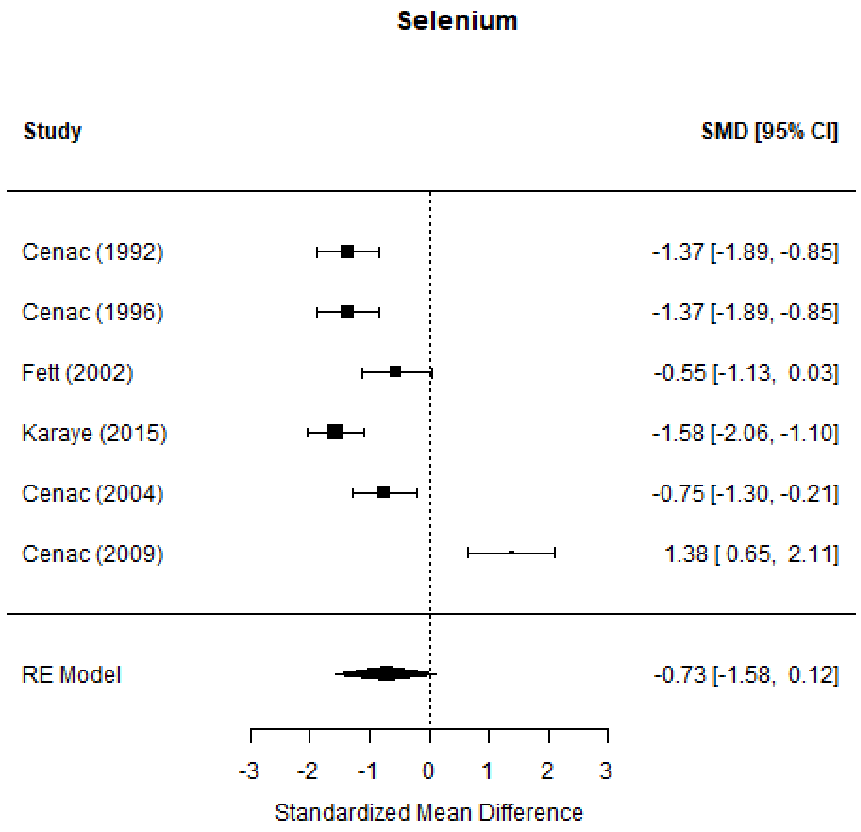

S.5 Forest plot of the SMD in Troponins between PPCM cases and healthy controls

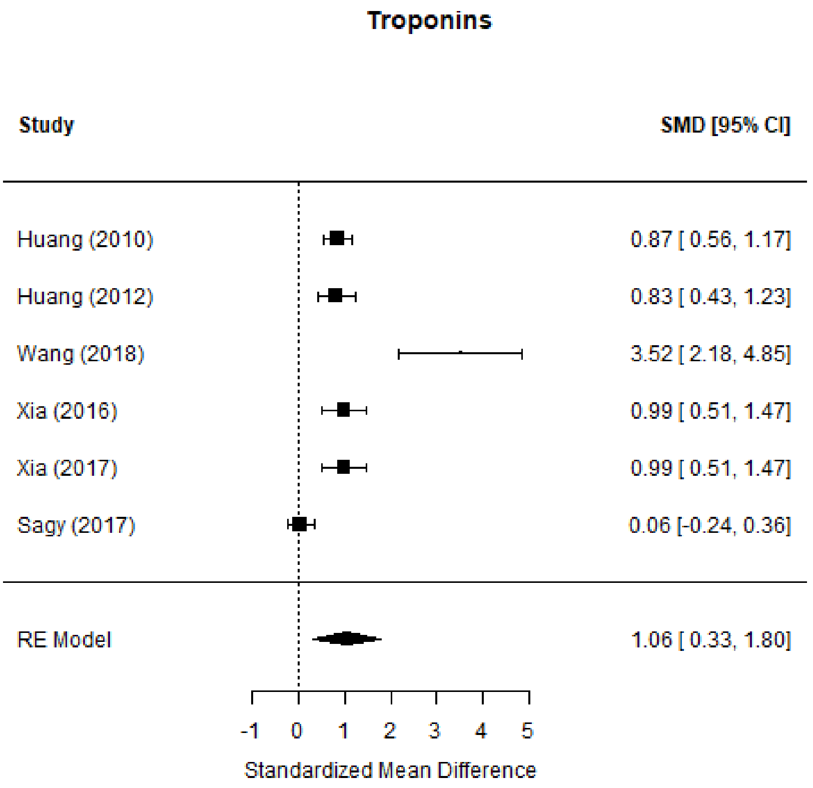

S.6 Forest plot of the SMD in Creatinine between PPCM cases and healthy controls

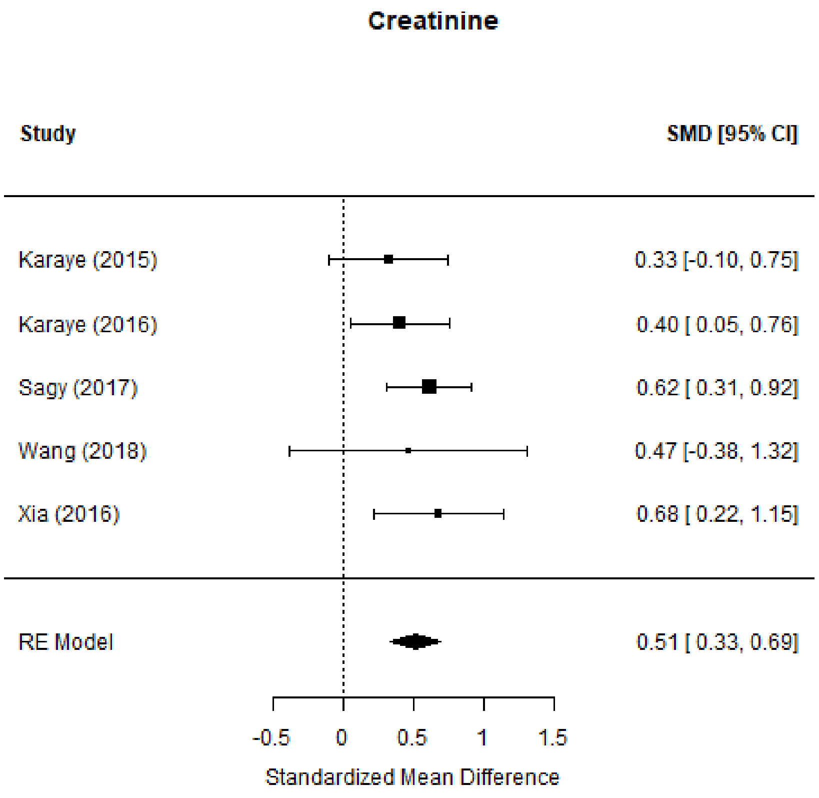

S.7 Forest plot of the SMD in White Blood Cells between PPCM cases and healthy controls

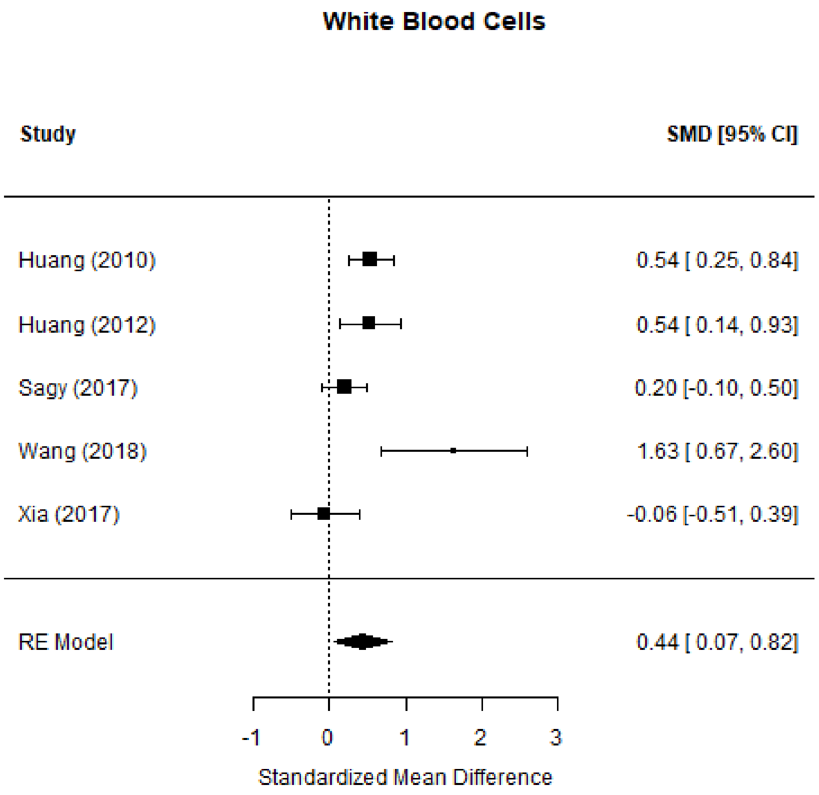

S.8 Forest plot of the SMD in Haemoglobin between PPCM cases and healthy controls

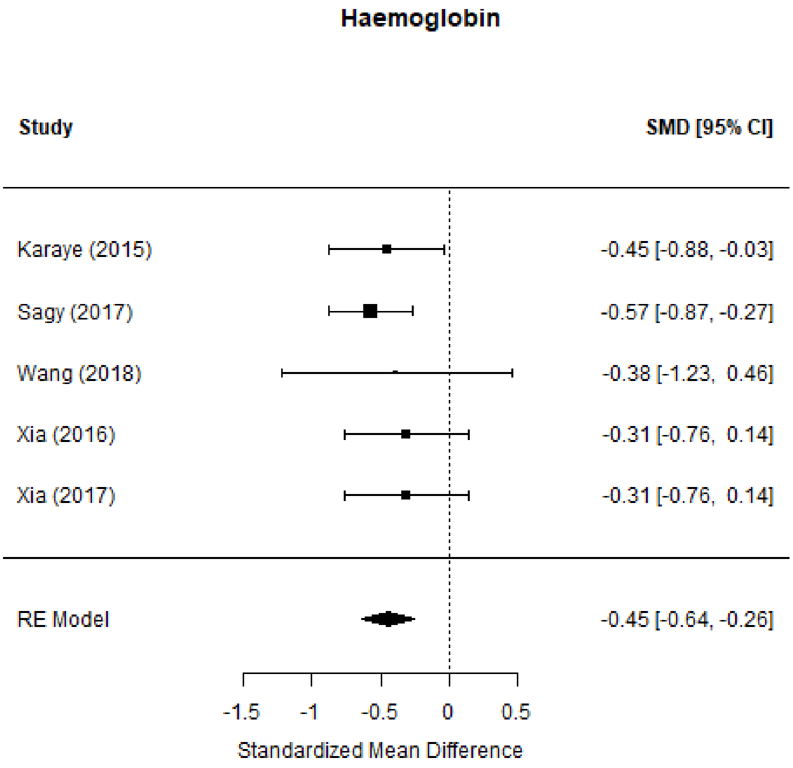

Supplement: Supplementary data [file openhrt-2020-001430supp005.pdf]
